# Magnitude-dependent response of osteoblasts regulated by compressive stress

**DOI:** 10.1038/srep44925

**Published:** 2017-03-20

**Authors:** Xiao-qing Shen, Yuan-ming Geng, Ping Liu, Xiang-yu Huang, Shu-yi Li, Chun-dong Liu, Zheng Zhou, Ping-ping Xu

**Affiliations:** 1 Department of Stomatology, Zhujiang Hospital, Southern Medical University, Guangzhou, China; 2 School of Dentistry, University of Detroit Mercy, Detroit, Michigan, USA; 3 Department of Oral and Maxillofacial Surgery, Guangdong Provincial Stomatological Hospital, Southern Medical University, Guangzhou, China; 4 Key laboratory of Oral Medicine, Guangzhou Institute of Oral Disease, Stomatology Hospital of Guangzhou Medical University, Guangzhou, China

## Abstract

The present study aimed to investigate the role of magnitude in adaptive response of osteoblasts exposed to compressive stress. Murine primary osteoblasts and MC3T3-E1 cells were exposed to compressive stress (0, 1, 2, 3, 4, and 5 g/cm^2^) in 3D culture. Cell viability was evaluated, and expression levels of *Runx2, Alp, Ocn, Rankl*, and *Opg* were examined. ALP activity in osteoblasts and TRAP activity in RAW264.7 cells co-cultured with MC3T3-E1 cells were assayed. Results showed that compressive stress within 5.0 g/cm^2^ did not influence cell viability. Both osteoblastic and osteoblast-regulated osteoclastic differentiation were enhanced at 2 g/cm^2^. An increase in stress above 2 g/cm^2^ did not enhance osteoblastic differentiation further but significantly inhibited osteoblast-regualted osteoclastic differentiation. This study suggested that compressive stress regulates osteoblastic and osteoclastic differentiation through osteoblasts in a magnitude-dependent manner.

Bone serves as the frame structure of the body to overcome gravity and provide rigid support in movement. To adapt to complicated mechanical stress, bone is under continuous remodeling: old or damaged bone is removed by osteoclasts and new bone is fabricated by osteoblasts. Bone mineral density is increased in athletes engaged in heavy physical activity^1^. Osteoporosis is one of the major health risks for astronauts living in low-gravity conditions^2^.

However, in such a mechanical stress-related process, the role of mechanical stress remains unclear. On the one hand, compressive stress is often introduced to induce bone resorption in dental practice. By inducing bone resorption around the roots, vertical compressive stress can intrude teeth into sockets, and horizontal compressive stress leads to teeth movement. This phenomenon is widely accepted as the biological basis of orthodontic treatment^3^. On the other hand, some neglected events lead to the opposite conclusion. For instance, compressive stress in teeth movement also enhances healing of bony defects^4^,^5^.

We previously reported that compressive stress induces resorption of maxillary bone in adult beagle dogs^6^. However, Deguchi *et al*. found that compressive stress enhances the osteogenesis of cortical bone around miniscrews and the miniscrew-bone contact rate^7^. These findings suggested that compressive stress may regulate activities of both osteoblast and osteoclast. This dual role coincides with the function of osteoblasts in bone remodeling: it transduces mechanical stimulus into biochemical signals that regulate osteoblastic and osteoclastic differentiation^8^. As a potential central regulator, osteoblasts are frequently discussed in studies on mechanical adaptive response of bone.

Unfortunately, *in vitro* studies showed contradictory results on the responses under compressive stress: inhibited/enhanced osteoblastic differentiation and inhibited/enhanced osteoblast-regulated osteoclastic differentiation have both been reported^9^,^10^,^11^,^12^. In studies that reported contradictory results using similar loading methods, magnitude was a major difference. A previous study reported that a compressive stress of 1.0 g/cm^2^ is optimal for osteoblastic differentiation^13^, but another study observed enhanced osteoblastic differentiation when compression was raised from 2.0 g/cm^2^ to 4.0 g/cm^2 ^11. Therefore, magnitude may be one of the parameters that determine the role of mechanical stress in bone remodeling.

In the present study, osteoblastic MC3T3-E1 cells and primary osteoblasts (POBs) of mouse calvaria were loaded with a series of compressive stress ranging from 0 g/cm^2^ to 5 g/cm^2^. The magnitude-dependent responses were evaluated with the perspectives of osteoblastic and osteoblast-regulated osteoclastic differentiation to provide novel interpretations into the role of mechanical stress in bone remodeling.

## Results

### Osteoblasts maintained the typical 3D growth pattern under compressive stress

**Figure 1:**
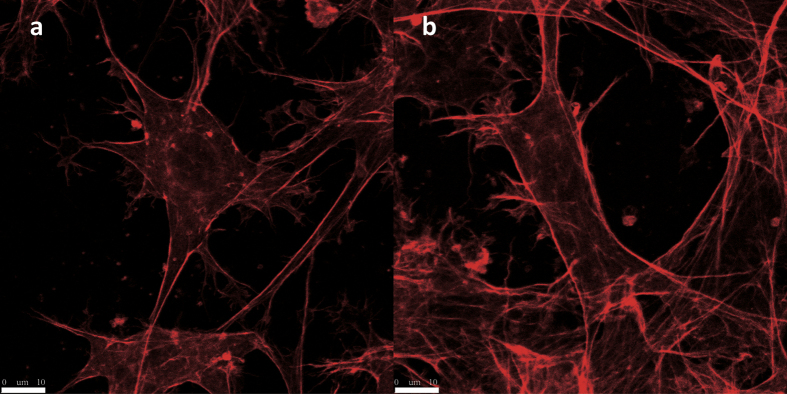
Confocal microscopy images of cells exposed to compressive stress. The cells were stained with rhodamine phalloidin. The images indicated that compressive stress did not eliminate typical 3D growth pattern in all groups. MC3T3-E1 cells exposed to 0 (**a**) and 5 g/cm^2^ (**b**) are presented (scale bar: 10 μm).

Among all the magnitudes used in this study, compressive stress did not eliminate the 3D growth pattern of osteoblasts in collagen gel. The MC3T3-E1 cells under compressive stress of 0 and 5 g/cm^2^ are shown in Fig. 1a and b.

### Cell viability was not inhibited by compressive stress

**Figure 2:**
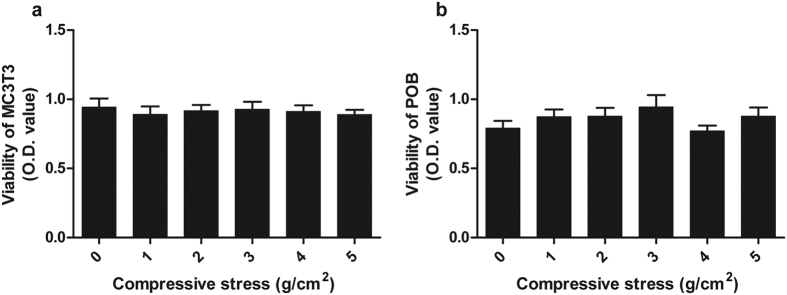
Viability of cells exposed to compressive stress. The viability of MC3T3-E1 cells (**a**) and POBs (**b**) was not inhibited by compressive stress ranging from 0 g/cm^2^ to 5 g/cm^2^. Data are displayed as the mean ± SD from one representative of three independent experiments (n = 5 per group). “p” denotes the significance level compared with the group exposed to 0 g/cm^2^ (control). *p < 0.05.

After application of compressive stress, the collagen gels containing cells were submerged into αMEM containing 10% FBS. Cell viability was determined using a Cell Counting Kit. No significant differences in cell viability were found among all groups (Fig. 2).

### Adaptive osteoblastic differentiation to compressive stress

**Figure 3:**
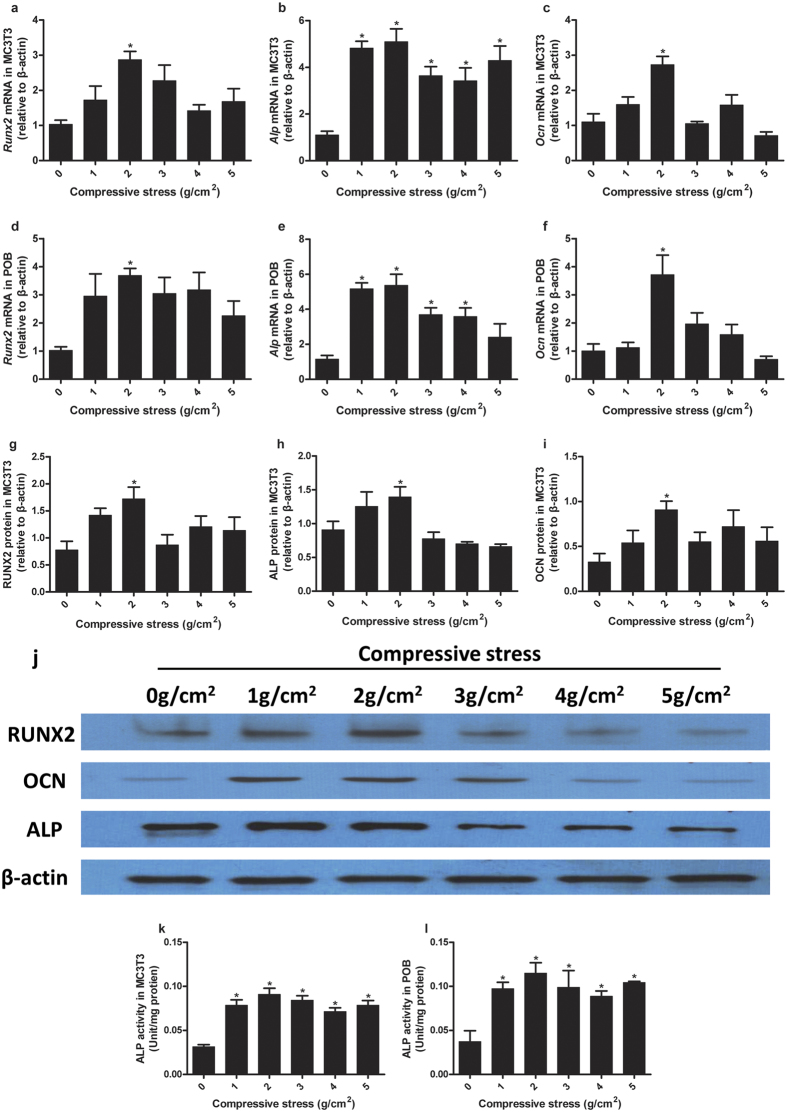
Adaptive osteoblastic differentiation to compressive stress. In MC3T3-E1 cells, *Runx2*, and *Ocn* mRNA increased significantly only when the compressive stress of 2 g/cm^2^ was applied (**a**,**c**). *Alp* mRNA increased significantly in all stress-loaded groups and the cells exposed to 2 g/cm^2^ and showed the highest average value (**b**). In POBs, *Runx2*, and *Ocn* mRNA expression also increased significantly in the group exposed to 2 g/cm^2^ (**d**,**f**). *Alp* mRNA expression increased significantly in all stress-loaded groups except 5 g/cm^2^, as well as showed the highest average value in the group exposed to 2 g/cm^2^, similar to MC3T3-E1 cells (**e**). The protein expression levels of RUNX2, ALP, and OCN in MC3T3-E1 cells were examined by Western blot. RUNX2, ALP, and OCN all increased significantly when a compressive stress of 2 g/cm^2^ was applied (**g**–**i**). Representative immunoblotting images are shown (**j**). With 14 days of continuous osteogenic induction, ALP activity increased significantly in MC3T3-E1 cells (**k**) and POBs (**l**) exposed to stress of any magnitude. The cells under compressive stress of 2 g/cm^2^ exhibited peak average values. Data are displayed as the mean ± SD from one representative of two (for Western blot) or three (for PCR and ALP activity) independent experiments (n = 5 per group). “p” denotes the significance level compared with the group exposed to 0 g/cm^2^ (control). *p < 0.05.

In MC3T3-E1 cells, *Runx2* mRNA expression showed a significant increase in the group exposed to compressive stress of 2 g/cm^2^. *Runx2* mRNA expression only slightly increased in other stress-loaded groups (1, 3, 4, and 5 g/cm^2^) and showed no significant differences with the control (Fig. 3a). *Alp* mRNA expression increased significantly in all stress-loaded groups, and the average value peaked in the group exposed to 2 g/cm^2^ (Fig. 3b). *Ocn* mRNA expression demonstrated a trend similar to *Runx2* (Fig. 3c). Meanwhile, protein expression was examined via Western blot (Fig. 3j). The protein expression of RUNX2, ALP, and OCN all increased significantly in the group exposed to 2 g/cm^2^ but showed no significant difference with the control in other stress-loaded groups (Fig. 3g,h and i).

In POBs, *Runx2*, and *Ocn* mRNA expression showed significant differences only in the group exposed to 2 g/cm^2^ (Fig. 3d and f). The mRNA expression of *Alp* increased significantly in all stress-loaded groups, except for the group exposed to 5 g/cm^2^, and showed the highest average value in the group exposed to 2 g/cm^2^ (Fig. 3e).

After application of compressive stress, the cells were further cultured in osteogenic medium for 14 days. Subsequently, ALP activity was tested. In both MC3T3-E1 cells and POBs, ALP activity increased significantly upon application of compressive stress, showing the highest average value in the group exposed to 2 g/cm^2^ (Fig. 3k and l).

### Adaptive osteoblast-regulated osteoclastic differentiation to compressive stress

**Figure 4:**
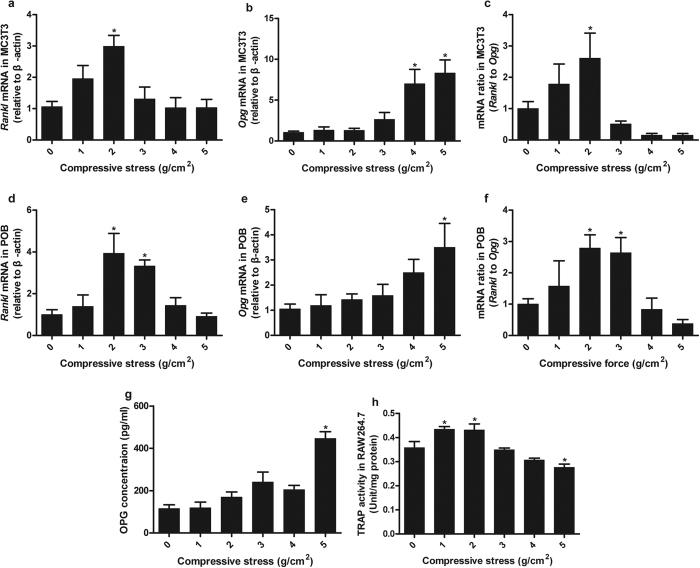
Adaptive osteoblast-regulated osteoclastic differentiation to compressive stress. In MC3T3-E1 cells, *Rankl* mRNA increased significantly only when a compressive stress of 2 g/cm^2^ was applied (**a**). *Opg* mRNA increased significantly in the groups exposed to 4 and 5 g/cm^2^ (**b**). The *Rankl/Opg* ratio increased significantly only in the group exposed to 2 g/cm^2^ (**c**). In POBs, *Rankl* mRNA increased significantly in the group exposed to 2 and 3 g/cm^2^ (**d**). *Opg* mRNA increased significantly in the group exposed to 5 g/cm^2^ (**e**). The *Rankl/Opg* ratio increased significantly in the groups exposed to 2 and 3 g/cm^2^ (**f**). The protein expression levels of RANKL and OPG in the culture medium of MC3T3-E1 cells were measured with ELISA. OPG expression showed a significant increase only in the cells exposed to 5 g/cm^2^ compressive stress (**g**). RANKL protein was not detected in the medium with ELISA. RAW264.7 cells were co-cultured with MC3T3-E1 cells exposed to compressive stress for 72 h with additions of 10^−8^ mol/L 1α,25(OH)_2_ D_3_ and 50 ng/mL M-CSF. TRAP activity increased significantly in the groups exposed to 1 and 2 g/cm^2^, but it was inhibited significantly in the group exposed to 5 g/cm^2^. Data are displayed as the mean ± SD from one representative of two (for ELISA) or three (for PCR and TRAP activity) independent experiments (n = 5 per group). “p” denotes the significance level compared with the group exposed to 0 g/cm^2^ (control). *p < 0.05.

In MC3T3-E1 cells, *Rankl* mRNA expression showed a significant increase only in the group exposed to compressive stress of 2 g/cm^2^ and no significant differences in other stress-loaded groups, but slight fluctuations existed (Fig. 4a). *Opg* mRNA expression increased significantly in the groups exposed to 4 and 5 g/cm^2^ (Fig. 4b). The *Rankl/Opg* gene expression ratio increased significantly in the group exposed to 2 g/cm^2^ (Fig. 4c). The protein expression levels of OPG and RANKL in the medium were determined by ELISA. OPG protein expression increased significantly in the group exposed to 5 g/cm^2^ (Fig. 4g), whereas RANKL protein was not detected in the medium by ELISA.

In POBs, *Rankl* mRNA expression showed significant increases in the groups exposed to 2 and 3 g/cm^2^ (Fig. 4d). *Opg* mRNA expression increased significantly only in the group exposed to 5 g/cm^2^ (Fig. 4e). The *Rankl/Opg* gene expression ratio increased significantly only in the groups exposed to 2 and 3 g/cm^2^ (Fig. 4f).

RAW264.7 cells were co-cultured with MC3T3-E1 cells under compressive stress for 72 h, and TRAP activity was tested. TRAP activity increased significantly in the groups exposed to 1 and 2 g/cm^2^, but it was inhibited in the group exposed to 5 g/cm^2^ (Fig. 4h).

## Discussion

Although the mechanical environment that osteoblasts experience *in vivo* is still under debate, cellular deformation caused by mechanical stress is believed to be essential in subsequent mechanotransduction^14^. Opposite responses of osteoblasts were induced with *in vitro* loading of compressive stress regarding the osteoblastic differentiation and osteoblast-regulated osteoclastic differentiation. In the present work, we tried to investigate the divergence by inducing the responses in a novel model.

In conventional *in vitro* loading models, compressive stress is applied by deforming cells with rigid planes^15^ or deforming elastic culture plates that bear cells^16^. In most cases, cells are cultured in a 2D monolayer and become severely deformed compared with their states *in vivo*, thereby increasing the inaccuracy of the study^17^. Given the intrinsic limitations of monolayer culture, 3D culture was employed to offer a novel perspective.

Collagen type I is a major extracellular matrix protein of bone and harbors several adhesive points for osteoblasts. It was used in 3D cell culture to mimic an *in vivo* environment. In this study, the typical 3D growth pattern of MC3T3 cells was observed under a confocal microscope: multiple processes originated from cell bodies and radiated in different directions to form an intercellular network with surrounding cells.

Tensile deformation perpendicular to compressive stress in gel soft as collagen is prominent because of Poisson’s effect. The combined deformation means that the cells in the same loading substrate experience different stress, which renders the parameter “magnitude” meaningless. Given that the tensile strain may induce unexpected cellular response^18^, the collagen gels used in our research were confined in closed space made of alginate calcium gel to minimize the accompanied tensile strain. As elastic modulus of alginate calcium gel is much higher than that of collagen gel, we can ignore the adverse influence of tensile strain and focus only on compressive stress loaded on cells. The model allowed us to investigate our research questions with compressive strain of designated magnitudes. No breaks were found in the two kinds of gels under compressive stress ranging from 0 g/cm^2^ to 5 g/cm^2^, and the collagen gels even maintained a certain thickness. The cells in the compressed gels showed similar 3D growth pattern to the non-compressed ones under confocal microscopic observation.

Osteoblasts transduce mechanical stimulus into biochemical signals through multiple mechanoreceptors, such as integrins^19^, pericellular tethers^20^, focal adhesions^21^, ion channels^22^, cadherins^23^, connexins^24^, and lipid rafts^25^. Transmitted via intracellular signaling cascades including MAPK^26^, PI3K/Akt^27^, GTPases^28^, Wnt^29^ and calcium^30^ pathways, the signals modulate osteoblastic differentiation by up-regulation of genes such as *Runx2, Alp*, and *Ocn*^31^. RUNX2 is a master control transcription factor of osteoblastic differentiation and function. ALP is usually observed at an early stage of osteoblastic differentiation and considered to reflect new bone formation. OCN is a bone-specific matrix protein expressed at the late stage of osteoblastic maturation. Osteoblasts also regulate osteoclastic differentiation via intercellular communications such as RANKL and OPG^32^. RANKL triggers osteoclastic differentiation by binding to its specific receptor RANK on osteoclast precursors. OPG is a secreted member of the tumor necrosis factor receptor family that binds to RANKL, blocking its interaction with RANK and inhibiting osteoclastic differentiation. The RANKL/OPG ratio is often used as an indicator of osteoclastic differentiation regulated by osteoblasts^33^.

In the present study, expression of *Runx2* and *Ocn* increased significantly at gene and protein levels in the group exposed to 2 g/cm^2^. ALP protein followed the same response to magnitude. However, *Alp* mRNA and ALP activity increased significantly in all stress-loaded groups. These results indicated that compressive stress ranging 0 to 5 g/cm^2^ enhances osteoblastic differentiation, and 2 g/cm^2^ is the optimal condition in this model. Above 2 g/cm^2^, the level of osteoblastic differentiation does not increase with the magnitude of stress. Meanwhile, in the group exposed to 2 g/cm^2^, increased expression of *Rankl* mRNA and *Rankl/Opg* gene expression ratio were observed in both MC3T3-E1 cells and POBs. The up-regulated osteoclastic differentiation was further demonstrated by increased TRAP activity in RAW264.7 cells co-cultured with MC3T3-E1 cells exposed to 2 g/cm^2^. As RANKL binds to RANK as a membrane-bound form^34^, it was not detected in the supernatants. Expression of *Opg* increased significantly at gene and protein levels when the stress increased to 5 g/cm^2^ and TRAP activity was inhibited at the same magnitude. These results indicated that compressive stress of 2 g/cm^2^ is the optimal condition for osteoblast-regulated osteoclastic differentiation in this model; but further increase of stress inhibits osteoclastic differentiation.

The results of the present study were in accordance with findings of earlier studies, if the responses were interpreted separately as enhanced osteoblastic differentiation and enhanced/inhibited osteoclastic differentiation. Several studies have shown that compressive stress can enhance osteoblastic differentiation within a certain range^35^,^36^. Sanuki *et al*. found that compressive stress induces osteoclastic differentiation via inhibited expression of OPG and elevated expression of M-CSF and RANKL in osteoblasts^37^. Meanwhile, Kaneuji *et al*. proposed that compressive stress inhibits osteoclastic differentiation via elevated expression of OPG in osteoblasts through the Wnt/Ca^2+^ pathway^9^. Collectively, these studies suggested the dual role of compressive stress in bone remodeling as a regulatory factor of osteoblastic and osteoclastic differentiation by acting on osteoblasts. Furthermore, the present study investigated the synchronous alterations in the two regulations to magnitude. The results revealed that the dual role of compressive stress may be dependent on magnitude.

Bone maintains its structural integrity to a certain degree in continuous remodeling. Such maintenance implies that the activities of osteoblasts and osteoclasts are coupled closely^38^. Although considerable signal molecules that enable communication between osteoblasts and osteoclasts have been identified, how the amount of resorbed bone determines the amount of bone formed remains unclear. In bone remodeling, bone cells are loaded with dynamic stress resulting from the continuous modulations of bone structure and mass. In the present study, the stress of different magnitudes could be termed as a partial simulation of dynamic loading *in vivo*. The magnitude-dependent responses of osteoblasts suggested that magnitude could be a crucial factor coupling osteoblasts and osteoclasts. Resorption weakens the integrity of bone, leading to increased stress loaded on osteoblasts. In response to the increased stress, bone formation is initiated to replace the resorbed bone. Osteoblasts are deactivated when the structural improvement of the bone matrix dissipates the stress. If the stress increases above a certain threshold, osteoclasts are inhibited protectively.

Although a biochemical mechanism about the inferred remodeling cycle still lacks comprehensive and systemic research, a small body of clinical evidence may support our hypothesis. In orthodontic treatment, stress induces bone resorption at the compression sites and bone formation at the tension sites. Continuous light forces are believed to be effective in producing efficient tooth movement, but simply increasing the force magnitude does not accelerate tooth movement^39^. Root resorption is less frequent when orthodontic force is increased, suggesting that osteoclastic activity is inhibited with excessive stress^40^.

In the present study, compressive stress that ranged from 0 g/cm^2^ to 5 g/cm^2^ was applied on MC3T3-E1 cells and POBs. The magnitude-dependent responses of osteoblasts were characterized by the expression levels of *Alp, Ocn, Runx2, Rankl*, and *Opg* at gene and protein levels; ALP activity in osteoblasts; and TRAP activity in osteoclast precursors co-cultured with osteoblasts. We proposed that compressive stress plays a dual role in bone remodeling, and stress magnitude is a key coupling factor between osteogenesis and osteoclastogenesis. In addition, our study provides an innovative *in vitro* compressive stress loading model for studies on cellular mechanotransduction.

## Methods

### Cell culture

Primary osteoblasts (POB) were isolated from the calvaria of newborn wild-type mice, as previously described^41^. In brief, pieces of calvaria were digested with 0.25% trypsin (Sigma, St. Louis, MO, USA) for 15 min and 0.1% type II collagenase (Sigma) for 30 min. The medium containing bone pieces was cultured for 5 days. Adherent cells were trypsinized and cultured in a new dish. The adherent cells on the new dish were used as primary osteoblasts. This experiment was performed in accordance with the guidelines provided by the Animal Care and Use Committee of Southern Medical University. The protocols were approved by the Animal Care and Use Committee of Southern Medical University (Guangzhou, China).

The murine osteoblastic cell line MC3T3-E1 subclone 14 and the murine monocyte/macrophage cell line RAW264.7 were obtained from the Cell Bank of the Chinese Academy of Sciences. The cells were cultured in α-minimal essential medium (αMEM; Gibco, Grand Island, NY, USA) supplemented with 10% fetal bovine serum (FBS; Gibco) and maintained at 37 °C in a humidified 5% CO_2_ atmosphere.

### Application of compressive stress

**Figure 5:**
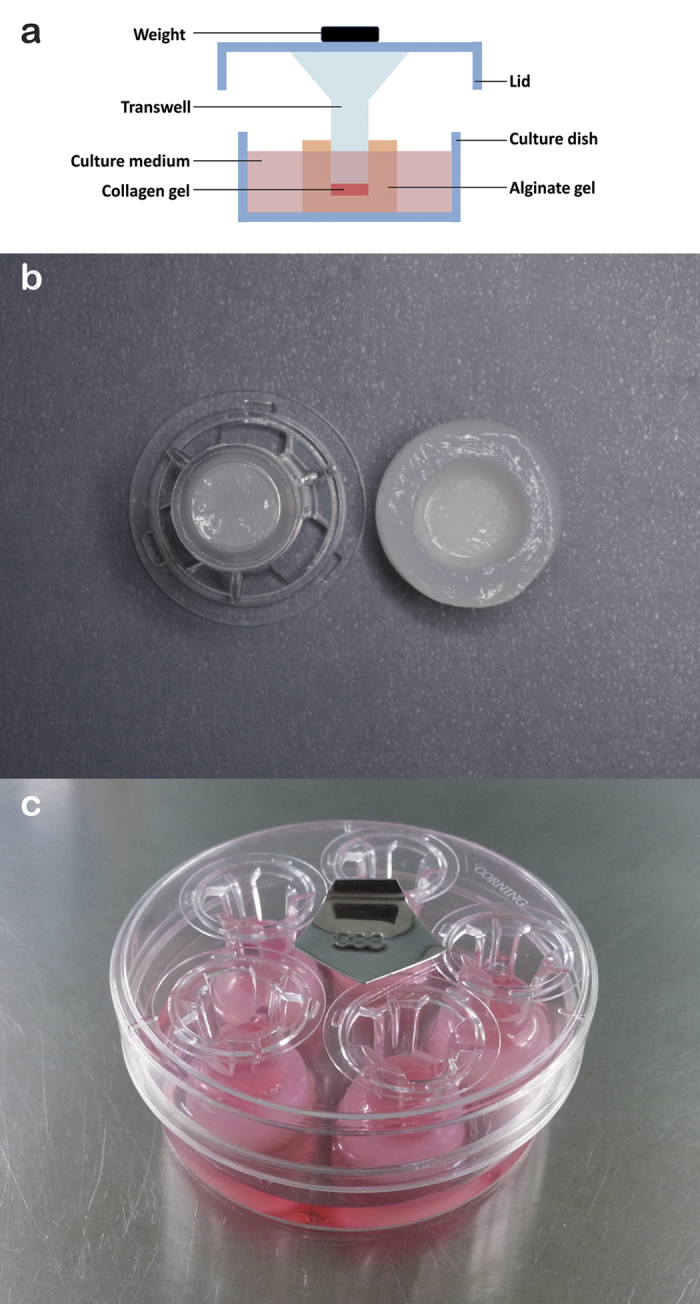
Compressive stress application model. Diagram of the model (**a**). The inner diameter of a customized well fitted with a Transwell insert (**b**). Every five wells were compressed together in one dish. The magnitude of compressive stress was adjusted by adding weights on the dish lid (**c**).

Compressive stress was applied to POBs and MC3T3-E1 cells in type I collagen-based 3D culture with Transwell inserts (pore size = 0.4 μm, Corning Incorporated, Corning, NY, USA; Fig. 5).

Customized culture well. One mL of 0.02 g/mL sodium alginate was added to each well of 24-well plates (Corning). Subsequently, 300 μL of 0.04 g/mL calcium chloride was added in the inner chamber of a Transwell insert as soon as it was placed in the well. After incubation for 24 h at 37 °C, the inserts were removed and the newly formed gels were used as the customized culture wells. The wells matched the shape of inserts perfectly (Fig. 5b).

3D culture. Cells were harvested from a monolayer culture and resuspended at a density of 10^7^/mL. Every 50 μL cell suspension was mixed with 700 μL of type I collagen solution (BD Biosciences, San Jose, CA, USA), 100 μL of 200 mM HEPES (4-(2-hydroxyethyl)-1-piperazineethanesulfonic acid) buffer (pH 7.5), 100 μL of 10 × αMEM, and 50 μL of 0.5 M sodium hydroxide solution. The mixture was added to the customized culture wells (200 μL/well). The mixture was allowed to polymerize for 1 h at 37 °C.

Application of compressive stress. The Transwell inserts were placed on the collagen gels, and 170 μL of osteogenic medium (αMEM with 10% FBS, 50 μg/mL ascorbic acid, and 10 mM β-glycerophosphate) was added in each inner chamber. The customized wells were placed in culture dishes, with five wells in one dish. Dish lids were then placed on the inserts. The magnitude of compressive stress was adjusted by adding weights on the lids (Fig. 5c). All the lids and inserts used were weighed before use, and their weights were considered independently. The collagen gels were exposed to compressive stress for 24 h at 37 °C. The following magnitudes of compressive stress were used: 0, 1, 2, 3, 4, and 5 g/cm^2^. In the control group, inserts were not placed and cells were exposed to compressive stress of 0 g/cm^2^. Please see more information in *Supplementary methods*.

### Co-culture of RAW264.7 cells with MC3T3-E1 under compressive stress

Type I collagen with MC3T3-E1 was introduced in the customized wells for polymerization. Subsequently, Transwell inserts were placed on the wells. RAW264.7 cells were seeded in the inner chambers (10^5^ cells/chamber), and MC3T3-E1 cells were subjected to compressive stress of 0, 1, 2, 3, 4, and 5 g/cm^2^. The cells were cultured in αMEM supplemented with 10% FBS, 1% penicillin–streptomycin (Beyotime, Shanghai, China), 10^−8^ mol/L 1α,25(OH)_2_ D_3_ (Sigma), and 50 ng/mL M-CSF (PeproTech, Rocky Hill, NJ, USA) for 72 h at 37 °C.

### Cell viability

After the application of compressive stress, the collagen gels were submerged into αMEM containing 10% FBS. Cell viability was measured using a Cell Counting Kit (Dojindo, Kumamoto, Japan) in accordance with the manufacturer’s instructions. Optical density was measured at 450 nm using a microplate reader (Multiskan EX, Thermo Fisher Scientific, Waltham, MA, USA).

### Microscopic observation

Collagen gels were fixed with 4% paraformaldehyde for 10 min at room temperature and then washed with PBS for 30 s. The cells were permeabilized in 0.1% TritonX-100 (Sigma) for 5 min at room temperature and then washed with PBS for 30 s. The cells were stained with 100 nM rhodamine phalloidin (Life Technologies, Eugene, OR, USA) for 30 min at room temperature and then washed with PBS for 1 min. Images were visualized using confocal microscopy (TCS SP8, Leica, Mannheim, Germany).

### Real-time polymerase chain reaction analysis

The harvested collagen gels were digested with Type I collagenase I (Sigma) for 1 h at 37 °C. The cells were collected with a centrifuge and washed with PBS. Total RNA was isolated using TRIzol Reagent (Invitrogen, Carlsbad, CA, USA). The concentration and purity of total RNA were measured using a spectrophotometer (NanoDrop 2000, Thermo Fisher Scientific). First-strand cDNA synthesis was conducted using HiScript^®^ II 1st Strand cDNA Synthesis Kit (Vazyme, Nanjing, China). For mRNA quantitation, an ABI 7500HT Fast Real-Time PCR System (Applied Biosystems, Foster City, CA, USA) was used with AceQ qPCR SYBR Green Master Mix (Vazyme). The primers used are listed in Table 1. β-Actin functioned as an endogenous control. The 2^−ΔΔCt^ method was used to calculate the relative expression levels.

### Western blot analysis

Collagen gels were collected and washed twice with PBS. Five gels exposed to the same stress were lysed together in ice-cold RIPA buffer. Total protein concentrations were measured with the BCA Protein Assay Kit (Thermo Scientific Pierce, Rockford, IL, USA). Protein samples (20 μg per lane) were separated by 10% SDS-PAGE gels, transferred to polyvinylidene fluoride membranes, and soaked for 2 h in a blocking solution (PBS buffer containing 5% nonfat dry milk and 0.1% Tween-20). Western blot was performed using the following primary antibodies: rabbit monoclonal to RUNX2 antibody (1:1,000, #8486, Cell Signaling Technology, Danvers, MA, USA), rabbit monoclonal to ALP antibody (1:10,000, ab108337, Abcam, Cambridge, MA, USA) rabbit polyclonal to OCN antibody (1:500, ab93876, Abcam), and mouse monoclonal to β-actin antibody (1:10,000, KM9001, Sungene Biotech, Tianjing, China). The secondary antibodies were goat anti-rabbit IgG(H + L)-HRP conjugated (1:5,000, LK2001, Sungene Biotech). Immunoblots were detected with an ECL kit (Thermo Scientific Pierce). β-Actin was used as an endogenous control. Quantity One (Bio-Rad, Hercules, CA, USA) was used for semi-quantitative analysis.

### ELISA

After the application of compressive stress, five pieces of alginate gel in the same dish were ground together in the medium. The mixtures were centrifuged. The concentrations of RANKL and OPG in the supernatant were determined using ELISA kits (R&D Systems, Minneapolis, MN, USA) in accordance with the manufacturer’s instructions.

### Alkaline phosphatase (ALP) activity

After the application of compressive stress, the collagen gels were collected from the customized culture wells using a microsurgical forceps. After culture in osteogenic medium for 14 days, the collagen gels were digested with 0.1% type I collagenase. The cells were harvested with a centrifuge and lysed in 0.5% Triton X-100. ALP activity was determined with an Alkaline Phosphatase Assay Kit (Beyotime) according to the manufacturers’ instructions. Protein concentrations were measured with the BCA Protein Assay Kit (Thermo Scientific Pierce). ALP activity was noted as King’s units/mg protein.

### Tartrate-resistant acid phosphatase (TRAP) activity

The RAW264.7 cells in inserts were lysed in 0.5% Triton X-100. TRAP activity was determined with a TRAP Kit (Beyotime) according to the manufacturers’ instructions. Protein concentrations were measured with a BCA Protein Assay Kit (Thermo Scientific Pierce). TRAP activity was noted as units/mg protein.

### Statistical analysis

Statistical analysis was conducted using IBM SPSS 22.0 version for Windows (IBM Corp, Armonk, NY, USA). All data were presented as mean ± SD. Statistical differences among the groups were assessed by one-way ANOVA. Post multiple comparisons were performed using Newman–Keuls test. The significant differences between stress-loaded groups and control (0 g/cm^2^) were labeled with asterisks. The significance level was set at p < 0.05.

## Additional Information

**How to cite this article:** Shen, X.- *et al*. Magnitude-dependent response of osteoblasts regulated by compressive stress. *Sci. Rep.* **7**, 44925; doi: 10.1038/srep44925 (2017).

**Publisher's note:** Springer Nature remains neutral with regard to jurisdictional claims in published maps and institutional affiliations.

## Supplementary Material

Supplementary Methods

## Figures and Tables

**Table 1:** Real-time polymerase chain reaction primers used in the experiments.

Target	Forward	Reverse	NCBI Ref. Seq.
*Alp*	TCCCACGTTTTCACATTCG	CGTCCACCACCTTGTAGCCA	NM_007431.3
*Ocn*	ACCATCTTTCTGCTCACTCTGC	CACTACCTTATTGCCCTCCTG	NM_007541.3
*Runx2*	TCAAGGGAATAGAGGGGATG	GGGAGGACAGAGGGAAACA	NM_001146038.2
*Rankl*	TACCTGTACGCCAACATTTGC	CGTGCTCCCTCCTTTCATCA	NM_011613.3
*Opg*	TGGCTGAGTGTTTTGGTGG	TGGTCTCTGTTTTGATGTTTCC	NM_008764.3
β-actin	ATGTGGATCAGCAAGCAGG	GTCAAAGAAAGGGTGTAAAACG	NM_007393.3
