# Supplementary material for: Magnitude-dependent response of osteoblasts regulated by compressive stress
Source: Sci Rep. 2017 Mar 20;7:44925. doi: 10.1038/srep44925 (PMC5357902; doi:10.1038/srep44925)
Supplement: Supplementary Methods [file srep44925-s1.pdf]

Magnitude-dependent response of osteoblasts regulated by compressive stress

Xiao-qing Shen [1,4 #], Yuan-ming Geng [1#], Ping Liu [2], Xiang-yu Huang [2], Shu-yi Li [3], Chun-dong Liu [1], Zheng Zhou [4*], Ping-ping Xu [2*]

1. Department of Stomatology, Zhujiang Hospital, Southern Medical University, Guangzhou, China
2. Department of Oral and Maxillofacial Surgery, Guangdong Provincial Stomatological Hospital, Southern Medical University, Guangzhou, China
3. Key laboratory of Oral Medicine, Guangzhou Institute of Oral Disease, Stomatology Hospital of Guangzhou Medical University, Guangzhou, China
4. School of Dentistry, University of Detroit Mercy, Detroit, Michigan, USA

#. These authors contributed equally to this work.

*. Corresponding author.

Correspondence to: Ping-ping Xu, No. 366 Jiangnandadao Road, Guangzhou China, 510280, Tel/Fax: 86-02084418626, Email: ppxu_smu@163.com. Zheng Zhou, 2700 Martin Luther King Jr. Blvd, Detroit, Michigan, 48208, Tel: 313-494-6667, Fax: 313-494-6666, Email: zhouzh1@unmercy.edu.

Supplementary methods

Design diagrams of the stress-application model

Application of compressive stress of 2-5g/cm^2. Each dish contained five alginate wells. Transwells were placed on the collagen gels. Dish lids were then placed on and contacted with the five Transwells simultaneously. The magnitude of compressive stress was adjusted by adding weights on the lids (Fig. S1a).

Application of compressive stress of 1g/cm^2. Abutments were added on the outer wall of the dishes. Each dish contained five alginate wells. The dish lids were placed on and supported by the abutments. The lids did not contact with the Transwells (Fig. S1b).

Application of compressive stress of 0g/cm^2. Each dish contained five alginate wells. No Transwells were added (Fig. S1c).

Application of compressive stress of 0g/cm^2 for co-culture. The alginate wells remained in the 24-well plates. Abutments were added on the edges of outer walls of the wells. Transwells were placed on and supported by the abutments (Fig. S1d).

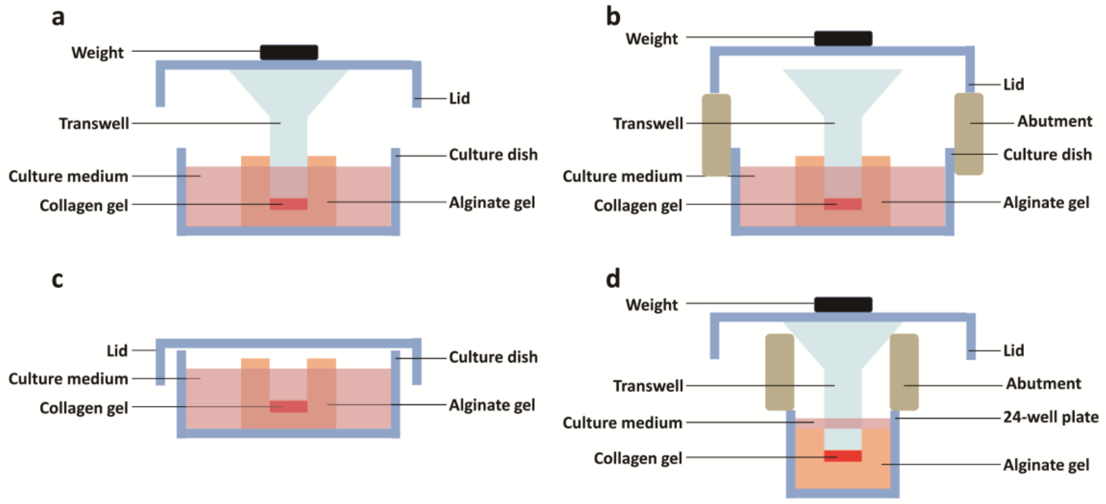

Fig. S1: Design diagrams of the stress-application model. Dish lids contacted with the Transwells in application of compressive stress of 2-5g/cm^2 (a). Dish lids did not contact with the Transwells in application of compressive stress of 1g/cm^2 (b). No Transwells were added in application of compressive stress of 0g/cm^2 (c). In application of compressive stress of 0g/cm^2 for co-culture, the alginate wells remained in the 24-well plates and the Transwells were supported by the abutments (d).

Application of compressive stress of designated magnitude

Calculation. The Transwells and lids of dishes were weighed before use. The calculation method is as follows.

$$Compressive\ stress = \frac{(\sum_{i=1}^{5}(W_{1i} + W_{2i})) + W_3 + W_4}{\sum_{i=1}^{5}\pi(\frac{d_i}{2})^2}$$

W_{1i}=weight of Transwell, W_{2i}=weight of culture medium in Transwell,
W_3=weight of lid, W_4=weight added,
d_i=outer diameter of Transwell

In application of compressive stress of 0g/cm^2, the collagen gels stayed in medium freely or just touched the Transwells slightly (in co-culture). W_1, W_2, W_3 and W_4 were zero in the condition. In application of compressive stress of 1g/cm^2, the collagen gels were compressed by the Transwells (W_1) and the medium in Transwells (W_2). Lids didn't touch the Transwells. W_3 and W_4 were zero in the condition.

Weight adjustment. The weight and outer diameter of Transwells and the weight of dish lids were slightly different between batches. The parameters were measured before use. The variations were counted in the calculation. An example of weight adjustment is shown (Tab. S1), supposing that the outer diameter of Transwell is 9.5mm, the weight of Transwell is 0.54g, the weight of lid is 2.90g, and the culture medium in Transwell is 170 ul (0.17g). The volume of the culture medium in Transwell could be adjusted in the range from 150ul to 200ul based on weights and

outer diameters of Transwells. The adjusted volume was then used in other groups. If the weights of wares deviated too far from the values in the example, another batch was used.

Table S1: An example of weight adjustment

Group	∑W1(g)	∑W2(g)	W3(g)	W4(g)	d (cm)	weight (g)	area (cm^2)	stress (g/cm^2)
control	0.00	0.00	0.00	0.00	0.95	0.00	3.54	0.00
1g/cm2	2.70	0.85	0.00	0.00	0.95	3.55	3.54	1.00
2g/cm2	2.70	0.85	2.90	0.65	0.95	7.10	3.54	2.00
3g/cm2	2.70	0.85	2.90	4.18	0.95	10.63	3.54	3.00
4g/cm2	2.70	0.85	2.90	7.72	0.95	14.17	3.54	4.00
5g/cm2	2.70	0.85	2.90	11.26	0.95	17.71	3.54	5.00

Preparation of abutments for dishes and 24-well plates

The abutments used in application of compressive stress of 1g/cm^2 (Fig. S2a & S2b) and in application of compressive stress of 0g/cm^2 for co-culture (Fig. S2c & S2d) and were prepared with light-curing resin (AP-X, Kuraray, Tokyo, Japan).

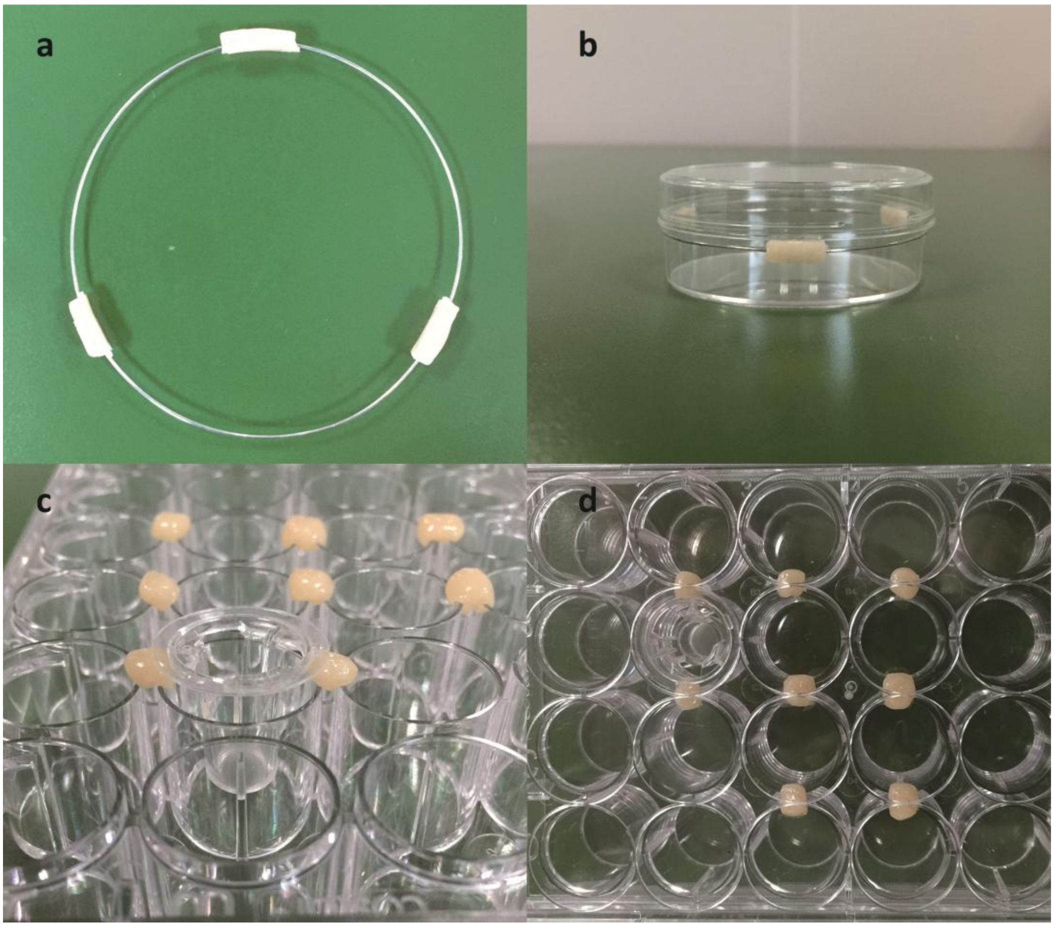

Fig. S2: Preparation of abutments. The abutments in application of compressive stress of 1g/cm^2 (a&b). They were made with resin and steel wires. The abutments in application of compressive stress of 0g/cm^2 for co-culture (c&d). They were added on the edges of outer walls of the wells in 24-well plates.
